# Release of β-galactosidase from poloxamine/α-cyclodextrin hydrogels

**DOI:** 10.3762/bjoc.10.330

**Published:** 2014-12-24

**Authors:** César A Estévez, José Ramón Isasi, Eneko Larrañeta, Itziar Vélaz

**Affiliations:** 1 Departamento de Química y Edafología, University of Navarra. C/ Irunlarrea s/n. 31008 Pamplona, Navarra, Spain

**Keywords:** controlled release, cyclodextrins, lactase, polypseudorotaxane, supramolecular gel

## Abstract

All mammals lose their ability to produce lactase (β-galactosidase), the enzyme that cleaves lactose into galactose and glucose, after weaning. The prevalence of lactase deficiency (LD) spans from 2 to 15% among northern Europeans, to nearly 100% among Asians. Following lactose consumption, people with LD often experience gastrointestinal symptoms such as abdominal pain, bowel distension, cramps and flatulence, or even systemic problems such as headache, loss of concentration and muscle pain. These symptoms vary depending on the amount of lactose ingested, type of food and degree of intolerance. Although those affected can avoid the uptake of dairy products, in doing so, they lose a readily available source of calcium and protein. In this work, gels obtained by complexation of Tetronic 90R4 with α-cyclodextrin loaded with β-galactosidase are proposed as a way to administer the enzyme immediately before or with the lactose-containing meal. Both molecules are biocompatible, can form gels in situ, and show sustained erosion kinetics in aqueous media. The complex was characterized by FTIR that evidenced an inclusion complex between the polyethylene oxide block and α-cyclodextrin. The release profiles of β-galactosidase from two different matrices (gels and tablets) of the in situ hydrogels have been obtained. The influence of the percentage of Tetronic in media of different pH was evaluated. No differences were observed regarding the release rate from the gel matrices at pH 6 (*t*_50_ = 105 min). However, in the case of the tablets, the kinetics were faster and they released a greater amount of 90R4 (25%, *t*_50_ = 40–50 min). Also, the amount of enzyme released was higher for mixtures with 25% Tetronic. Using suitable mathematical models, the corresponding kinetic parameters have been calculated. In all cases, the release data fit quite well to the Peppas–Sahlin model equation, indicating that the release of β-galactosidase is governed by a combination of diffusion and erosion processes. It has been observed that the diffusion mechanism prevails over erosion during the first 50 minutes, followed by continued release of the enzyme due to the disintegration of the matrix.

## Introduction

In the Western diet, carbohydrates contribute about 50% of calories, distributed in the following ratio: starch (50%), sucrose (30%), lactose (6%), maltose (1–2%), and others (12%: trehalose, glucose, fructose, sorbitol, cellulose, hemicellulose and pectin). Lactose, sucrose and maltose constitute a significant proportion, especially in infants, whose sole or main source of food is milk [1]. Disaccharides, such as lactose, must be hydrolyzed in order to be absorbed by the human body. For absorption to occur, lactose must be attacked by substrate-specific enzymes present in the digestive system, resulting in the production of monosaccharides, which are readily absorbed through the intestinal membrane. The intolerance to lactose is a clinical syndrome related to the intake of lactose characterized by the presence of some of the following symptoms: abdominal pain, diarrhea, nausea, flatulence and/or bloating. Symptoms can vary in each individual depending on the amount of lactose ingested, the degree of intolerance and the type of food consumed [2]. Notably, within this terminology, lactose malabsorption is defined as a pathophysiological phenomenon, resulting in insufficient lactose absorption, because the intake of the disaccharide is greater than the capacity for the hydrolysable action of the lactase enzyme (i.e., a lactase deficiency). The most radical treatment for lactose intolerant patients is to avoid products containing this disaccharide, or to reduce the intake according to the level of intolerance. After taking such precautions, the symptoms usually disappear; however, milk and dairy products are a concentrated source of calcium and other nutrients (e.g., high quality proteins, riboflavin) responsible for the growth and strengthening of bones in children and for preventing bone demineralization in adults.

Lactase supplements marketed in the form of capsules provide only limited relief because oral lactase tablets disintegrate in the stomach and only a portion of the ingested enzyme survives the unfavorable pH and enzymatic conditions of the upper gastrointestinal tract [3]. β-Galactosidase (lactase) is a tetrameric enzyme that contains four identical chains of 1021 amino acids each [4]. The mechanism involves transferring the D-galactose moiety of a lactose molecule to an acceptor with a hydroxy group. When the galactosyl acceptor is water, a galactose molecule is produced and the hydrolysis of lactose occurs. However, this transfer can be performed onto other acceptors such as sugars, yielding oligosaccharides through a transglycosylation mechanism [5].

Hydrogels are 3D networks capable of absorbing considerable amounts of water, and are suitable for a variety of pharmaceutical and biomedical applications, such as drug delivery and tissue engineering. In particular, hydrogels are excellent vehicles for proteins due to their high water content, good compatibility, and controllable release kinetics [6]. The hydrophilic nature of the hydrogels is due to the presence of groups along the polymer chains such as hydroxy, carboxy, amide and sulfonic acid [7]. The cross-linking may be either physical or chemical (covalent). According to the reversible and tunable nature of self-assembly, the physically cross-linked hydrogels have recently received attention as potential injectable and encapsulation materials.

Poloxamers (Pluronics) are triblock copolymers comprised of polyethylene oxide (PEO) and polypropylene oxide (PPO) blocks. Poloxamines (Tetronics) are x-shaped copolymers comprised of four diblock PEO–PPO copolymer chains attached to a central ethylenediamine group [8]. Their sensitivity to pH due to the central group offers interesting features as compared with the micelles of non-ionic block copolymers (such as Pluronics). Namely, with respect to the development of pharmaceutical nanocarriers, they have the ability to hold/release drugs as a function of pH [9].

**Figure 1:**
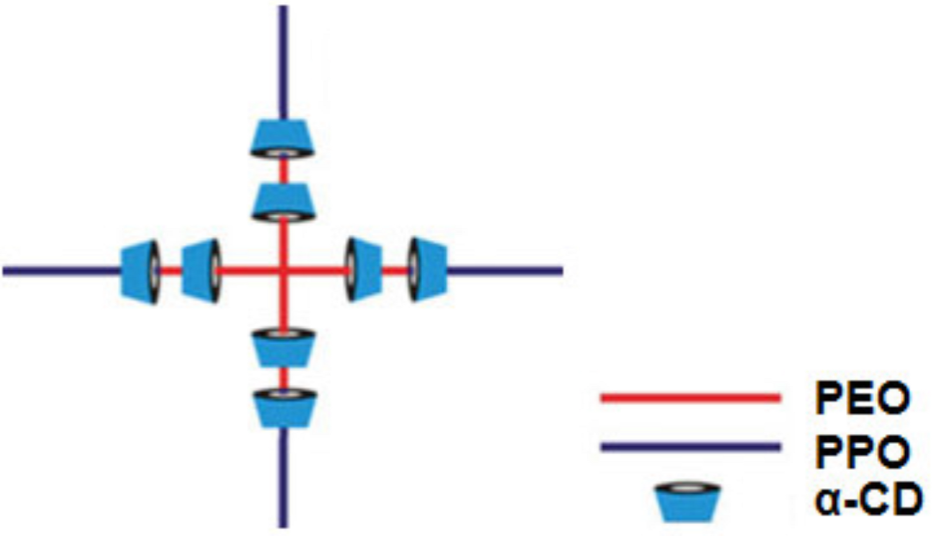
Inclusion complex (polypseudorotaxane) between α-cyclodextrins and reverse Tetronics.

Cyclodextrins (CD) are macrocyclic compounds consisting of several glucose units linked by α-D-1,4-glycosidic bonds. Despite their high solubility in water, the internal cavity of cyclodextrins is non-polar and these compounds are able to form host–guest complexes by the inclusion of hydrophobic molecules. Cyclodextrins also act as hosts in the formation of inclusion compounds with polymer chains through non-covalent interactions [10]. For instance, incorporation of 5% α-CD transforms dilute Tetronic solutions into gels, which can be used as supramolecular drug delivery systems [11]. Thus, cyclodextrin-based physical hydrogels assembled through microcrystalline formation are based upon the aggregation of polypseudorotaxane structures [12]. In the case of reverse Tetronics, α-cyclodextrin (composed of six glucose units) forms a polypseudorotaxane structure in water with the inner PEO units of these copolymers, as can be seen in Figure 1. We have recently shown that these structures form self-assembled gels under certain conditions [13].

These gels can be designed to encapsulate and then continuously release the lactase protein for efficient digestion of lactose in the body. For pharmaceutical applications, surface-eroding polymer matrices are interesting because in these systems, drug release follows zero order kinetics [14]. This erosion feature provides an advantage in many applications compared to other non-eroding systems because biocompatible polymers are gradually absorbed by the body with no need for a subsequent surgical removal. However, these systems can also present some disadvantages depending on the nature of the degradation products, which can be toxic, immunogenic or carcinogenic [15].

In this work, our aim is to determine the effectiveness of self-assembled, α-cyclodextrin/90R4 poloxamine gels and tablets for the controlled release of β-galactosidase at different pH values.

## Results and Discussion

### Complex formation between lactase, α-CD and 90R4

The supramolecular complex formation between the polyethylene oxide–polypropylene oxide (PEO–PPO) copolymer blocks of Tetronic 90R4 and α-CD resulted in a white viscous fluid. The rate of formation depends on the concentration of the 90R4 copolymer, α-CD and water. The relatively slow kinetics can be attributed to the arrangement of the blocks in the copolymer. α-CD must surpass the outer PPO blocks to reach the PEO blocks and form stable structures known as (pseudo)polyrotaxanes. PEO chains and oligoethylenes of different molecular weights form inclusion complexes with α-CD. On the other hand, the wider PPO chains yield inclusion complexes with β and γ-CD. For reverse poloxamine 90R4, the enthalpy-driven complexation between α-CD and inner PEO blocks makes it possible to overcome the energy barrier of α-CD sliding over the relatively bulky PPO blocks [16].

**Figure 2:**
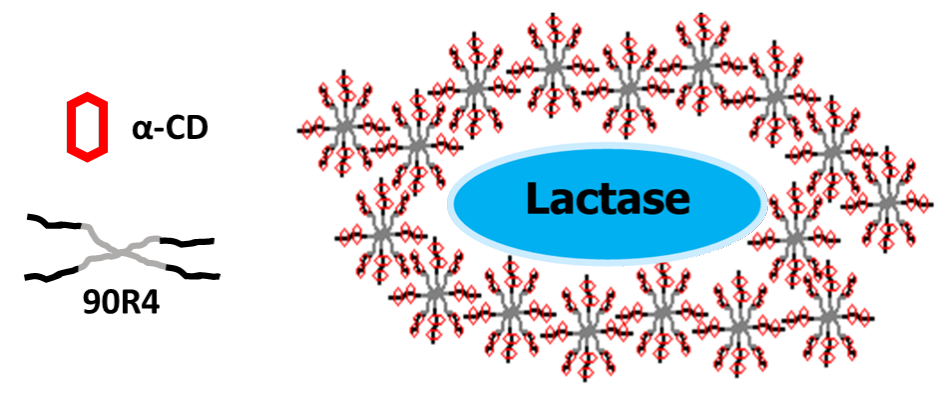
90R4/α-CD gels loaded with lactase. Octablock Tetronic molecules (grey) are threaded by CDs (red) forming a supramolecular assembly that entraps the protein molecules (blue).

Lactase molecules are entrapped within the supramolecular cross-linked gels which are formed by the addition of aqueous, protein-loaded α-CD to Tetronic 90R4. The outer PPO blocks of the copolymer interact forming micelles, while inner PEO blocks form inclusion complexes with α-CD. These moieties are also capable of interacting by hydrogen bonding, contributing to the stability of the network structure (Figure 2).

In a previous work, the rheological behavior of poloxamine/α-CD mixtures was investigated [16]. A 10% (w/w) solution of α-CD was selected as an intermediate value between the solubility limit of this cyclodextrin (14%) and a lower limit which yields mostly solutions instead of gels (7% and below). Two different reverse Tetronic amounts were tested, 25% and 15%. Table 1 shows the compositions of the two different types of 90R4/α-CD gels evaluated in this study, namely T25a10 (Table 1, entry 1) and T15a10 (Table 1, entry 2), respectively.

**Table 1:** Composition of the gels used in this work.

Entry	Gel name	Composition (%)		

		Tetronic 90R4	α-CD	Water

1	T25a10	25	10	65
2	T15a10	15	10	75

Considering a stoichiometric copolymer/CD ratio, 20% (w/w) of the copolymer corresponds to four CD molecules per poloxamine molecule (i.e., a single α-CD molecule per PEO complex block). As shown in our previous work [16], once each PEO block is threaded to one cyclodextrin moiety (on average) the conformation of the Tetronic arms change, making the self-assembly process feasible. In addition, X-ray diffraction showed that some degree of order can be found in the gel structure, attributable to the formation of EO/α-CD complexes that become aggregated into ordered domains.

**Figure 3:**
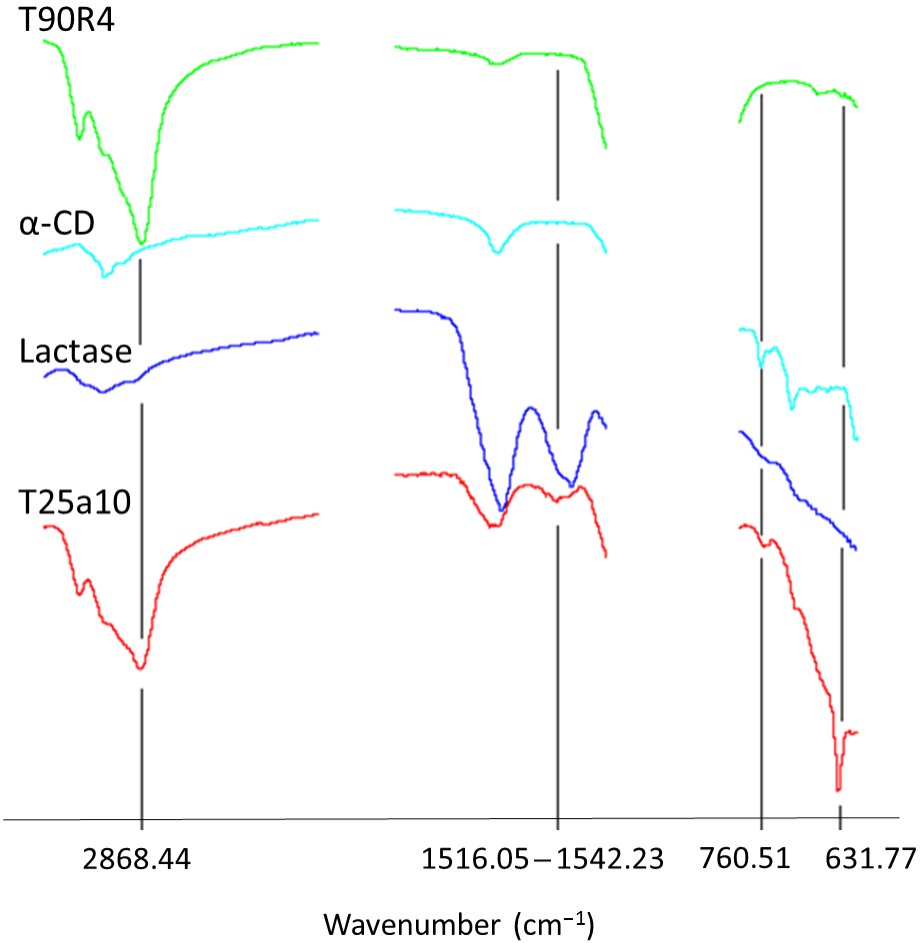
FTIR spectra of Tetronic 90R4, α-CD, lactase and T25a10 complex.

As can be seen in Figure 3, the FTIR spectrum of T25a10 loaded with lactase shows the contributions of all its components. The carbon–carbon bonds of poloxamine Tetronic 90R4 appear at 2860–2880 cm^−1^. Additionally, α-CD has a characteristic band in the fingerprint region, which is also present in the complex, next to the amide peak of lactase. Finally, the complex shows a new band that originates at 650 cm^−1^ and can be attributed to the formation of the inclusion complex between α-CD and Tetronic 90R4. For a physical mixture of Tetronic 90R4 and α-CD, the band at 650 cm^−1^ is not present, indicating that the inclusion complex is not formed.

### Analysis of the release of lactase from gels and tablets

**Figure 4:**
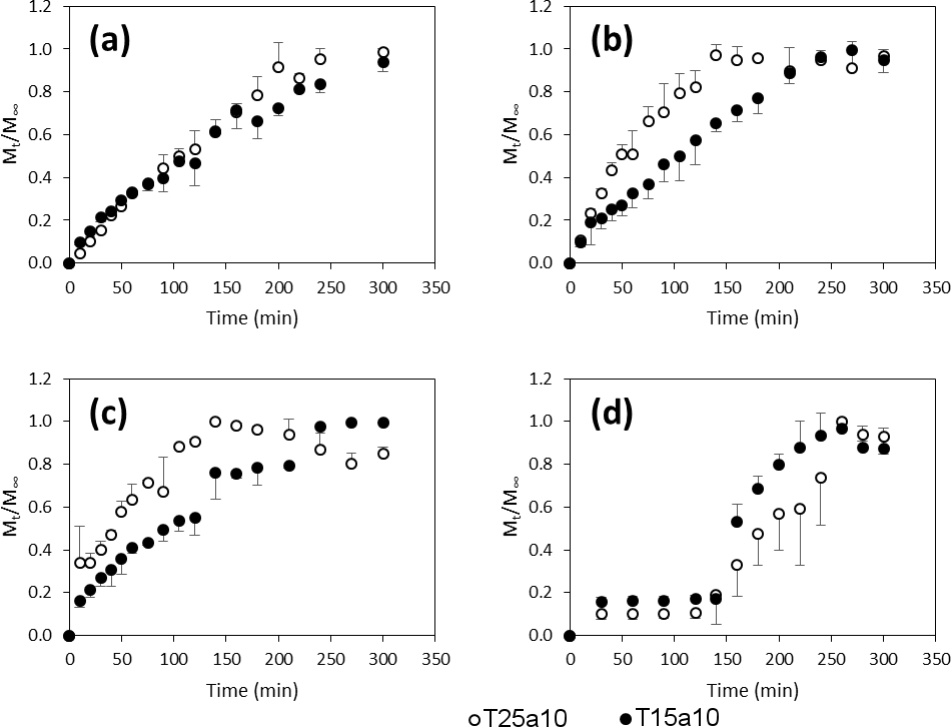
Release profiles of lactase from T25a10 (open circles) and T15a10 (filled circles) at pH 6 from a) gels with 29452 ALU, b) tablets with 4000–4500 ALU, c) tablets with 500–900 ALU and d) tablets with 4000–4500 ALU from pH 1.2 to pH 6 (d). ALU: acid lactase units.

Figure 4 shows the release profiles of lactase from gels and tablets at pH 6 and 37 °C. The normalized fraction of lactase released at time *t*, related to the maximum release (M_∞_) is shown for a better comparison. It was observed that both the gels and tablets are gradually dissolved over the course of time. At the end of the release experiments, clear solutions were obtained in all cases, except for the tablets in the pH study, for which heterogeneous mixtures were obtained. Figure 4a shows that release of lactase from T15a10 and T25a10 gels demonstrate similar profiles, while Figure 4b and Figure 4c show that the release of the enzyme from T25a10 tablets are faster than those from T15a10. In contrast, Figure 4d indicates that the opposite is observed when the pH is changed from 1.2 to 6. A greater protection of the enzyme by a higher load of Tetronic 90R4 occurs, and the corresponding release is slower in this case.

From the data collected in the release experiments, different parameters have been calculated. The mean dissolution time (MDT) of the matrix, the dissolution efficiency (DE) of the active ingredient, the time for which one half of the active ingredient has been released (*t*_50_) from tablets and gels, and the relationship between the amount released and the actual amount loaded (R/L) are shown in Table 2.

**Table 2:** Release parameters of gels and tablets loaded with lactase.

Entry	Sample	Type	pH	MDT (min)	*t*_50_ (min)	DE (%)	R/L (%)

1	T25a10	Gel	6	196	105	73.3	79.9
2		Tablet^a^	6	102	50	58.1	98.9
3		Tablet^b^	6	114	40	77.9	100
4		Tablet^c^	1.2–6	204	180	62.9	46.4
5	T15a10	Gel	6	155	105	71.7	74.7
6		Tablet^a^	6	129	90	62.8	91.9
7		Tablet^b^	6	125	90	65.9	83.6
8		Tablet^c^	1.2–6	189	160	60.5	28.9

^a^Tablet containing 4000–4500 ALU (lactase units), ^b^tablet containing 500–900 ALU, ^c^tablet containing 4000–4500 ALU with change of pH in medium.

For the T25a10 gel, both the mean dissolution time (MDT) and the half-release time, *t*_50_ are long compared to the tablets of the same composition (Table 2, entries 1–3). The same trend is observed for the T15a10 complexes. There is a small difference between the MDTs of T15a10 and T25a10 gels, however, the *t*_50_ values are the same for both (*t*_50_ = 105 min, Table 2, entry 1 and entry 5). For the tablet, both the MDT and *t*_50_ values for T25a10 tablets are shorter than those for T15a10, which indicates a faster release from T25a10 for neutral pH values (Table 2, entries 2, 3, 6 and 7).

The ratio between the amount of lactase released and that initially loaded (R/L) also indicates that the T25a10 gel and tablets are more effective for the release of the enzyme in a neutral medium. An acidic dissolution medium for tablets resulted in a loss of enzymatic activity of the lactase. This is evidenced in Table 2 (entries 4 and 8), where release assays were performed in two different pH media: 1.2 for the first 2 hours and 6.0 for the remaining 3 hours. The R/L ratio for the T25a10 tablet was 46% (Table 2, entry 4), whereas that of T15a10 was approximately 29% (Table 2, entry 8), indicating that Tetronic 90R4 protects the enzyme more effectively at a higher concentration. Taking into account that the release of lactase is faster in the case of T25a10 (Figure 4b), it is obvious that even after release into the medium, the enzyme molecules can be protected by an excess of eroded poloxamer molecules from the tablet. Nevertheless, the values obtained for R/L, especially in the case of T25a10, indicate that the enzyme could perform its desired action in the intestine after being subjected to the acidic pH environment of the stomach. Both tablets (Table 2, entries 4 and 8) have the highest MDT and *t*_50_ values. This is due to the acidic pH 1.2 (during the first 2 hours) conditions, in which most of the enzyme released is denatured.

A comparative study between the effectiveness of the tablets prepared in this investigation and two types of commercial chewable tablets (3500 and 4500 ALU/tablet) was conducted from pH 1.2 to pH 6, similar to that reported in the Experimental section.

A high lactase activity was observed in the samples under neutral pH; however, it is known that before reaching the enterocytes of the duodenum, the enzyme must survive the acidic environment of the stomach for a period of approximately two hours. Our studies indicate that crushed chewable tablets would not be able to exert their action after facing an acidic solution. In contrast, the T25a10 and T15a10 tablets showed resistance to an acidic medium and a controlled release of the enzyme in a neutral medium (pH 6). Thus, they are predicted to be more effective for lactose intolerant patients, even when the partial denaturation of the enzyme released in the acidic environment is taken into account (Table 2).

The lactase activity of commercial tablets showed enzyme deactivation in acidic medium due to protein denaturation. This phenomenon is favored when the tablet is crushed (chewable tablets) because their coating is removed and the surface area of the polymer increases causing increased contact with the medium. Thus, the lactase activity data in acidic media for the commercial tablets could not be collected.

The type of drug, its polymorphism, crystallinity, particle size and other factors can influence the release kinetics. A water-soluble drug incorporated into a matrix typically utilizes a diffusion mechanism for release, whereas for a poorly soluble active ingredient, the main mechanism of release is through erosion of the matrix [17–18]. The models used to ascertain the release mechanisms in this study were Korsmeyer–Peppas, Higuchi, Peppas–Sahlin, zero order, first order, and Hopfenberg.

Korsmeyer developed a simple formula to describe the release of the active pharmaceutical ingredient (API) from a polymeric system. They found that this model is acceptable up to an amount of 60% of API released. The exponent of the power law indicates the release mechanism; thus, for the case of cylindrical tablets, 0.45 ≤ *n* corresponds to a diffusion mechanism that obeys Fick’s law, while 0.45 < *n* < 0.89 indicates non-Fickian transport. Finally, if *n* ≥ 0.89 the model points to a relaxation phenomenon of the polymer matrix (erosion) [19].

Higuchi’s model explains the release of an API from a permeable matrix when the drug loading is much higher than its solubility limit [20]. This model can be applied to the analysis of non-erodible matrices such as hydrogels, where API release can be described as a simple diffusion process that follows Fick’s law [19]. The mathematical model developed by Peppas and Sahlin combines both the Fickian and the non-Fickian contributions of API release, that is, it uses both the diffusion and erosion terms. Finally, the release of APIs from erodible systems with various geometries was analyzed by Hopfenberg.

The results of the analysis of the release profiles of lactase from gels and tablets are shown in Supporting Information File 1. By applying the Korsmeyer–Peppas model to gels and tablets at pH 6, exponent values between 0.5 and 1 were obtained, indicating a possible contribution from both polymer erosion and diffusion. Likewise, similar values were obtained for the pre-exponential constant, indicating that the processes involved are very similar. This was not the case for the release from tablets in the pH-shift experiments: initially, in acidic media, the constants were different for both gels (T25a10 and T15a10), and at higher pH, the exponent indicates relaxation or complete erosion of the matrix.

**Figure 5:**
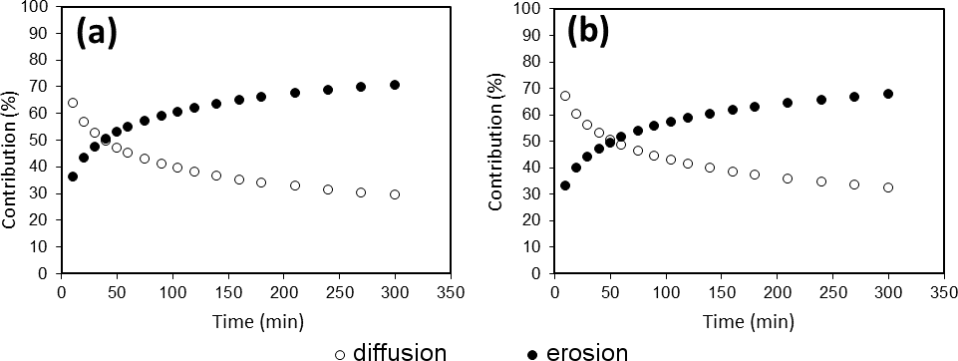
Contribution of diffusion (open circles) and erosion (filled circles) mechanisms from polymers a) T25a10 and b) T15a10 regarding the delivery of lactase.

Poor correlation coefficients resulted in all instances when testing Higuchi, zero order or first order equations, and thus these cannot be used to describe the release profiles. On the other hand, the Peppas–Sahlin equation yields the best correlation coefficients, fitting the release profiles of lactase in all cases. Next, in order to assess the relative influence of the diffusion and erosion mechanisms, kinetic constants of diffusion and erosion at pH 6 were obtained for T15a10 and T25a10 tablets, as shown in Figure 5.

As can be seen, the erosion mechanism dominates the diffusion release of lactase from T15a10 and T25a10 matrices after the first 50 minutes. Although lactase molecules located on the outside of the tablet diffuse easily, as the experiment continues, the polymer matrix relaxes so that the lactase inside the matrix is released by erosion. A similar result was found in previous work for the release of bovine serum albumin from poloxamine matrices [21], although in that case, erosion predominates after 5 h.

### Effect of storage time and matrix composition on the activity of lactase

The lactase activity results obtained in several release experiments from different matrices indicate that the matrix does not affect the enzymatic activity of the protein during a five hour test at pH 6. Values close to 100% activity were found in all cases. The average values calculated for the enzyme released relative to the initial amount loaded for α-CD, Tetronic 90R4, and pulverized T25a10 tablets were 98.7 ± 6.0, 94.5 ± 6.8 and 97.4 ± 8.8, respectively.

Note that even in the case of a mixture of the lactase protein with an amphiphilic block copolymer (such as Tetronic 90R4), the activity is as high as 95%. In this case, the block copolymer does not induce protein denaturation. Moreover, it has been found that poloxamers can have a positive effect on folding, since they suppress aggregation of denatured proteins [22].

With respect to storage time, the activity for pure lactase was compared to that of freshly prepared lactase tablets (T15a10 and T25a10) dissolved in a neutral medium (pH 6) and those after three months of storage time. The results show higher losses in the activity for the T25a10 polymer. More than 20% efficiency is lost due to a 90 day storage time in this case, while for the T15a10 sample, a decrease in activity of less than 10% is found. In contrast, pure lactase enzyme loses 7% activity in that period of time.

## Conclusion

In this study, the lactase enzyme is surrounded by the inclusion complex assemblies formed between Tetronic and α-CD, and is released from the polymer matrix mainly by erosion of the matrix. According to the Peppas–Sahlin model, the contribution of diffusion is considerably lower than that of the erosion mechanism. The poloxamine/cyclodextrin gels and tablets were shown to be capable of controlled release of the enzyme over an extended period of time. Additionally, the effectiveness of the tablets after a storage period of three months was evaluated, and indicated that the activity decreased (as expected), but less so for the formulation with a low amount of poloxamine. Notably, the tablets present a protective effect against an acidic pH environment with increased poloxamine content. Since a pH change greatly affects the activity of the lactase enzyme, the best formulation of a matrix must also consider this aspect in order to improve its applicability in the pharmaceutical field.

## Experimental

### Materials

**Figure 6:**
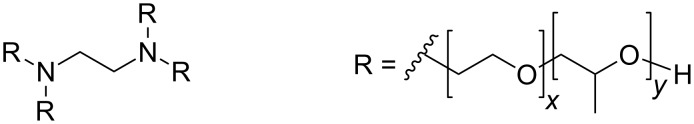
Chemical structure of a reverse Tetronic.

β-D-Galactosidase, with an enzymatic activity of 73654 acid lactase units per gram (ALU/g), was donated by Laboratorios Salvat S.A. (Spain). α-Cyclodextrin (α-CD), from Wacker Chemie AG (water content approximately 8%), was used as received. The poloxamine Tetronic 90R4 (Sigma-Aldrich) was a yellowish, viscous liquid (3870 cP at 25 °C) with an average molecular weight *M*_90R4_ ≈ 7200 g/mol (as given by the manufacturer). The chemical composition was determined by ^1^H NMR: PO_16_EO_18_ per arm (Figure 6).

Dissolution media were prepared using type I deionized water (Millipore Elix 3, minimum resistivity at 25 °C, 18 MOhm, electrical conductivity <0.1 µS/cm), hydrochloric acid (37%, Panreac, Spain), dibasic sodium phosphate and monobasic potassium phosphate (99% purity, Panreac, Spain).

In order to determine the activity of the β-D-galactosidase (lactase), 2-nitrophenyl-galactopyranoside (ONPG) (Sigma-Aldrich, ≥98% purity, 301.3 g/mol) was used as a substrate to simulate the action of the enzyme on lactose and its dissociation. The ONPG substrate requires a slightly acidic medium for enzyme lactase activity, therefore a buffer (pH 4.5) was used according to USP. Sodium hydroxide, acetic acid, and sodium carbonate (Panreac, Spain) were used as reagents.

### Methods

**Preparation of self-assembled gels loaded with lactase:** Two types of gels (T15a10 and T25a10) were prepared by mixing the appropriate amounts of α-CD, Tetronic 90R4, and water. α-CD and lactase were dissolved in water, and then mixed with the poloxamine under constant stirring to form the gels. The mixtures were kept overnight at room temperature prior to analysis. Gels were directly prepared in the vessel where the dissolution kinetics assays were performed (dissolution tests, see below). T15a10 and T25a10 gels contained 15% and 25% of Tetronic 90R4, respectively, and 10% α-CD each. 20 g of each gel were loaded with 400 mg of lactase (29462 ALU) for the release experiments.

**Preparation of tablets loaded with lactase:** In order to prepare tablets whose lactase content is similar to that of commercial capsules, water was removed from the gels. After each gel was frozen at −50 °C, it was lyophilized (Cryodos Telstar). The tablets were compacted using a Perkin Elmer hydraulic press. The resulting diameter of the tablets was 12.9 mm and the thickness was between 4.6 and 5.1 mm. Two sets of 500 mg tablets with different lactase contents were prepared. The first (T25a10a and T15a10a) had a high load of lactase, ≈4000–4500 ALU, and the second set (T25a10b and T15a10b) had a lower amount, ≈500–900 ALU.

For the characterization of the complexes, a Nicolet Avatar 360 FTIR Specac (Thermo Electric, Inc.) infrared spectrophotometer with a resolution of 4 cm^−1^, a DTGS detector and OMNI 7.2 software was used. Spectra were obtained in the region from 4000–600 cm^−1^, using a MKII Golden Gate ATR (attenuated total reflectance) device.

**Determination of enzymatic activity of lactase:** A calibration curve was obtained from a stock solution which contains 1 unit of lactase per mL. With this (according to the manufacturer), the activity of pure lactase was defined as 73654 ALU/g, and the concentration needed to achieve 1 unit/mL was 1.36 × 10^−5^ g/mL. The results show a good linearity (correlation coefficient, R^2^ = 0.9981) for the absorbance (ABS = 1.1053 [LACT] + 0.0203), where the concentration of the protein [LACT] is expressed in lactase units per milliliter of solution (ALU/mL).

The official monograph USP 37 assay, used to determine lactase activity of preparations derived from *Aspergillus oryzae*, is based on hydrolysis of the substrate *o*-nitrophenyl β-D-galactopyranoside (ONPG) at 37 °C and pH 4.5 (buffer prepared using acetic acid and sodium hydroxide) for 15 minutes. ONPG (370 mg) was dissolved in 100 mL of buffer immediately before the analysis since ONPG is photolabile and decomposes after 2 hours. 500 mL of each sample were added to 2 mL of the ONPG solution. After exactly 15 min, 2.5 mL of 10% sodium carbonate was added to stop the reaction. The amount of yellowish *o*-nitrophenol (ONP) produced was analyzed by spectrophotometry (Agilent 8453 UV–vis spectrophotometer) at 416 nm.

**Lactase release kinetics in vitro – dissolution tests:** The release from tablets and the erosion of gels was evaluated using dissolution test equipment (SOTAX AT 7 Smart USP, meets the specifications of USP37–NF32) [23], with a bath temperature of 37 °C and a paddle stirring speed of 100 rpm. Initial release studies of both gels and tablets were performed in 500 mL of a pH 6 phosphate buffer solution (optimal for lactase activity), taking 2 mL samples at different times; sample volumes were replaced with fresh medium. The lactase enzyme activity of the collected samples was analyzed according to the corresponding official USP monograph USP37–NF32, in duplicate for the gels and in quadruplicate for the tablets.

An additional study was performed to assess the resistance of the tablets at pH 1.2 (gastric) and the release at pH 6 (intestinal). After sampling for two hours in an acidic medium (HCl 0.1 M), 400 mL of phosphate buffer was added to reach pH 6 and the release of lactase was analyzed for 3 hours according to USP 37 (in quadruplicate for the two types of tablets).

In the case of commercial chewable tablets, six of each type were pulverized and the amount corresponding to one tablet was used as a representative sample in each experiment. The assays were made in duplicate.

**Effect of storage time and matrix composition on the activity of lactase:** To evaluate the effect of the matrix on lactase activity, a T25a10 tablet was pulverized and dissolved in 500 mL of phosphate buffer (pH 6) at 37 °C and 100 rpm in the dissolution test equipment. Samples were taken every 30 minutes for 5 hours and the activity was evaluated. In addition, duplicate tests were performed with mixtures of lactase and α-CD and mixtures of lactase and Tetronic 90R4 to ascertain the possible influence of each component of the complex. Furthermore, the influence of storage time on enzyme activity was evaluated for T25a10 and T15a10 tablets after 3 months of storage in a desiccator.

## Supporting Information

File 1 Full analysis of release profiles for tablets and gels loaded with lactase using different kinetic equations.
