# Supplementary material for: Release of β-galactosidase from poloxamine/α-cyclodextrin hydrogels
Source: Beilstein J Org Chem. 2014 Dec 24;10:3127–35. doi: 10.3762/bjoc.10.330 (PMC4311648; doi:10.3762/bjoc.10.330)
Supplement: File 1 — Full analysis of release profiles for tablets and gels loaded with lactase using different kinetic equations. [file Beilstein_J_Org_Chem-10-3127-s001.pdf]

Supporting Information

for

Release of β-galactosidase from poloxamine/α-cyclodextrin hydrogels

César A. Estévez, José Ramón Isasi, Eneko Larrañeta, Itziar Vélaz*

Address: Departamento de Química y Edafología, University of Navarra. C/ Irunlarrea s/n. 31008 Pamplona (Navarra, Spain).

Email: Itziar Vélaz - itzvelaz@unav.es

*Corresponding author

Full analysis of release profiles for tablets and gels loaded with lactase using different kinetic equations

Table S1. Analysis of release profiles for tablets and gels loaded with lactase with different kinetic equations (using ≤ 80% of data)

sample	Type	pH	Korsmeyer-Peppas			Higuchi		Zero order		First order		Peppas-Sahlin				Hopfenger	
			$k_{kp}*10^2$ (min^{-n})	n	R^2	k_H*10^2 ($min^{-1/2}$)	R^2	$k_{zo}*10^3$ (min^{-1})	R^2	$k_{PO}*10^3$ (min^{-1})	R^2	k_D*10^2 (min^{-m})	k_E*10^3 (min^{-2m})	m	R^2	$k_{HP}*10^1$ ($ALU*min^{-1}*mm^{-2}$)	R^2
**T25a10**	Gel	6	1.7 (±0.1)	0.72 (±0.04)	0.990	4.8 (±0.3)	0.867	4.6 (±0.1)	0.989	6.8 (±0.3)	0.968						
	Tablet a	6	1.2 (±0.6)	0.69 (±0.04)	0.986	7.2 (±0.2)	0.951	6.8 (±0.5)	0.948	13.7 (±0.3)	0.993	2.6 (±1.0)	12.0 (±1.7)	0.43	0.985	29.3 (±0.9)	0.977
	Tablet b	6	1.4 (±2.0)	0.69 (±0.06)	0.955	8.0 (±0.2)	0.960	6.8 (±0.8)	0.874	17.1 (±1.4)	0.907	8.9 (±1.6)	3.4 (±2.7)	0.43	0.997	4.9 (±0.4)	0.830
	Tablet c	1.2	7.9 (±0.6)	0.04 (±0.02)	0.999	10.8 (±1.2)	0.779	0.7 (±0.1)	0.412	1.2 (±0.3)	0.315	3.3 (±0.3)	2.5 (±0.4)	0.43	0.984	1.7 (±0.3)	0.292
		6	8.0 0.6	0.47 (±0.07)	0.946	7.2 (±0.5)	0.479	0.7 (±0.1)	0.960	4.8 (±0.9)	0.531	17.9 (±4.2)	24.9 (±4.3)	0.43	0.985	5.2 (±0.6)	0.668
**T15a10**	Gel	6	1.9 (±0.3)	0.70 (±0.03)	0.986	4.9 (±0.1)	0.943	3.5 (±0.2)	0.960	6.6 (±0.2)	0.981						
	Tablet a	6	1.5 (±0.2)	0.76 (±0.02)	0.994	5.2 (±0.2)	0.926	4.1 (±0.1)	0.986	7.2 (±0.2)	0.983	1.4 (±0.3)	7.8 (±0.5)	0.43	0.995	16.4 (±0.4)	0.984
	Tablet b	6	8.0 (±0.8)	0.64 (±0.06)	0.952	5.5 (±0.1)	0.959	4.5 (±0.4)	0.934	8.3 (±0.4)	0.956	3.5 (±1.1)	6.1 (±1.6)	0.43	0.996	3.2 (±0.2)	0.906
	Tablet c	1.2	10.8 (±0.4)	0.04 (±0.01)	0.999	10.9 (±1.2)	0.772	0.7 (±0.2)	0.409	2.1 (±0.4)	0.331	5.3 (±0.4)	4.2 (±0.5)	0.43	0.989	2.7 (±0.6)	0.291
		6	17.6 1.6	0.37 (±0.04)	0.964	10.7 (±0.9)	0.488	0.5 (±0.1)	0.864	6.4 (±1.2)	0.492	5.9 (±2.9)	14.6 (±3.0)	0.43	0.993	3.6 (±0.4)	0.625

Load of lactase in tablets: (a) (4000-4500 ALU), (b) (500-900 ALU), (c) (4000-4500 ALU) pH (1.2 to 6)

Korsmeyer-Peppas (KP)

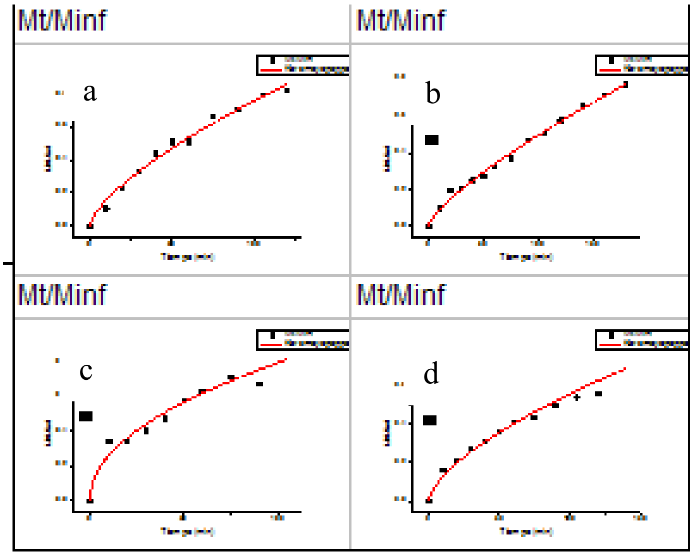

Figure S1. KP release profiles for tablets at pH 6: (a) T25a10 (4000-4500 ALU), (b) T15a10 (4000-4500 ALU), (c) T25a10 (500-900 ALU), (d) T15a10 (500-900 ALU)

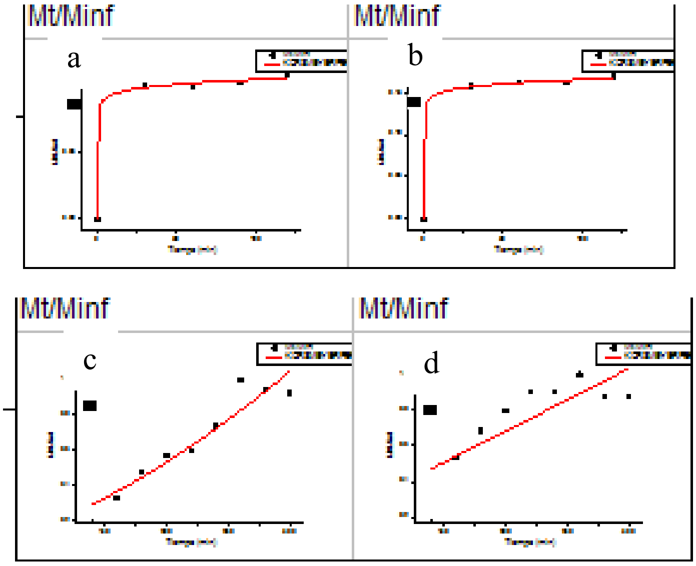

Figure S2. KP release profiles (4000-4500 ALU) from tablets with pH change: (a) T25a10 pH 1.2, (b) T15a10 pH 1.2, (c) T25a10 pH 6, (d) T15a10 pH 6

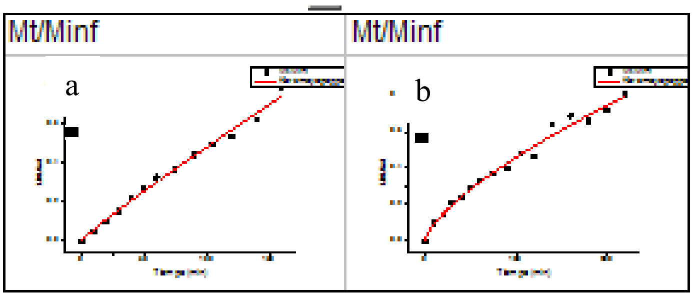

Figure S3. KP release profiles from gels at pH 6 (29450 ALU): (a) T25a10, (b) T15a10

Higuchi (H)

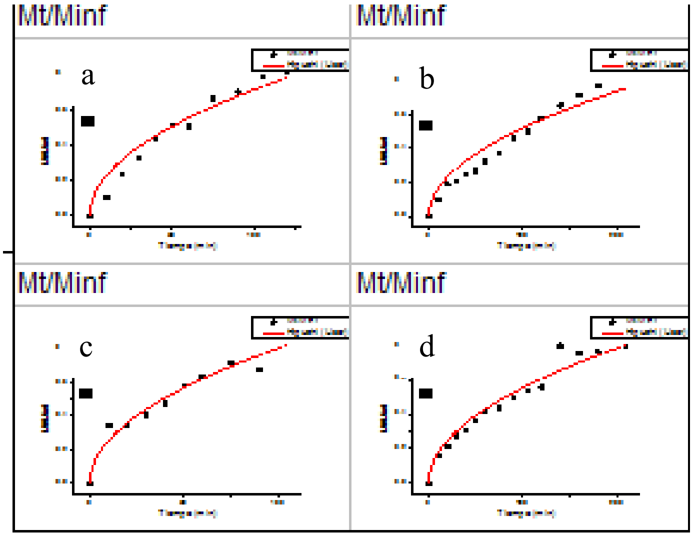

Figure S4. H release profiles for tablets at pH 6: (a) T25a10 (4000-4500 ALU), (b) T15a10 (4000-4500 ALU), (c) T25a10 (500-900 ALU), (d) T15a10 (500-900 ALU)

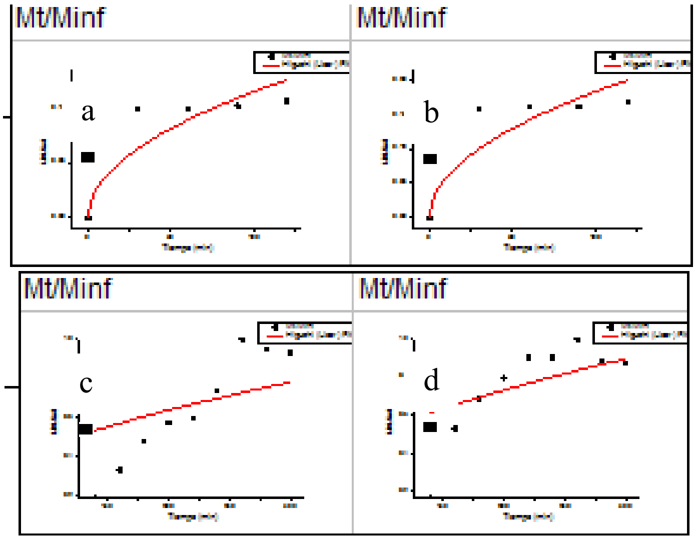

Figure S5. H release profiles (4000-4500 ALU) from tablets with pH change: (a) T25a10 pH 1.2, (b) T15a10 pH 1.2, (c) T25a10 pH 6, (d) T15a10 pH 6

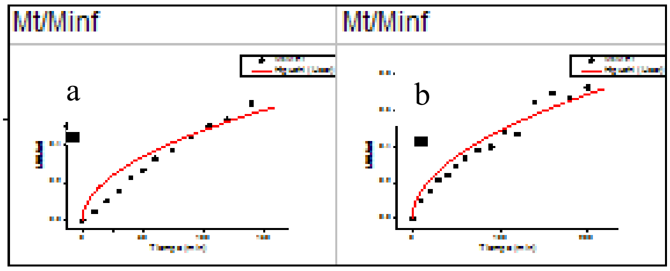

Figure S6. H release profiles from gels at pH 6 (29450 ALU): (a) T25a10, (b) T15a10

Zero order (ZO)

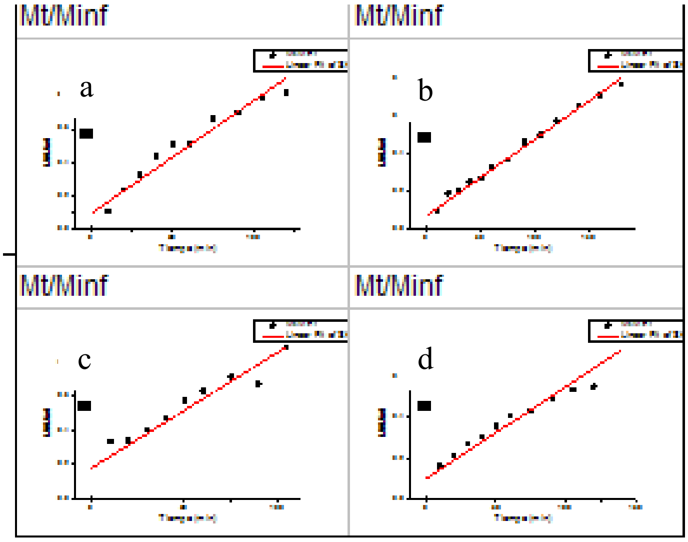

Figure S7. ZO release profiles for tablets at pH 6: (a) T25a10 (4000-4500 ALU), (b) T15a10 (4000-4500 ALU), (c) T25a10 (500-900 ALU), (d) T15a10 (500-900 ALU)

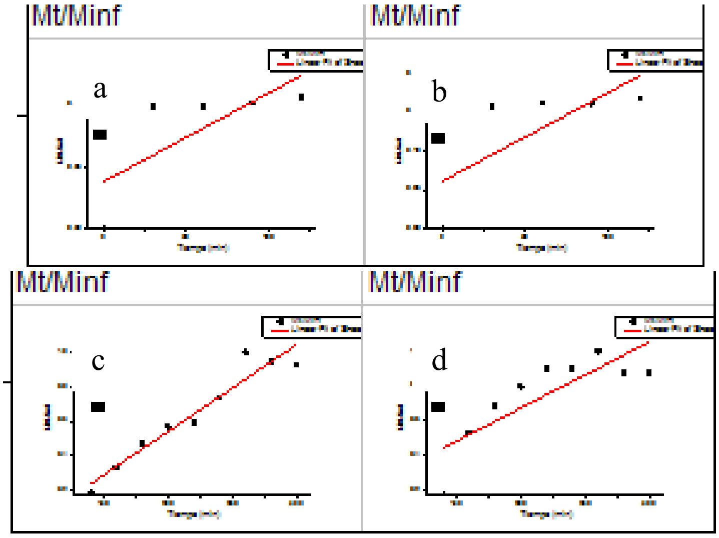

Figure S8. ZO release profiles (4000-4500 ALU) from tablets with pH change: (a) T25a10 pH 1.2, (b) T15a10 pH 1.2, (c) T25a10 pH 6, (d) T15a10 pH 6

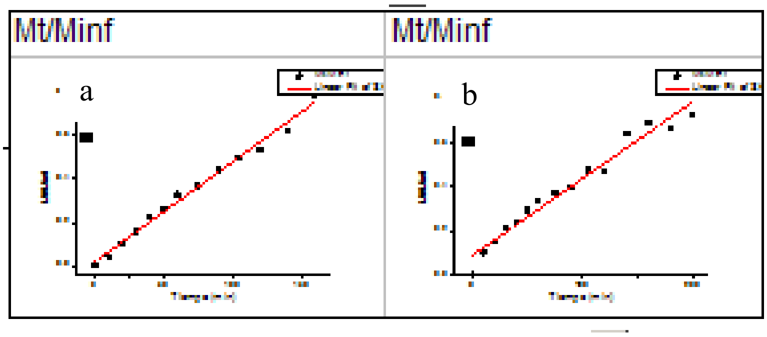

Figure S9. ZO release profiles from gels at pH 6 (29450 ALU): (a) T25a10, (b) T15a10

First Order (FO)

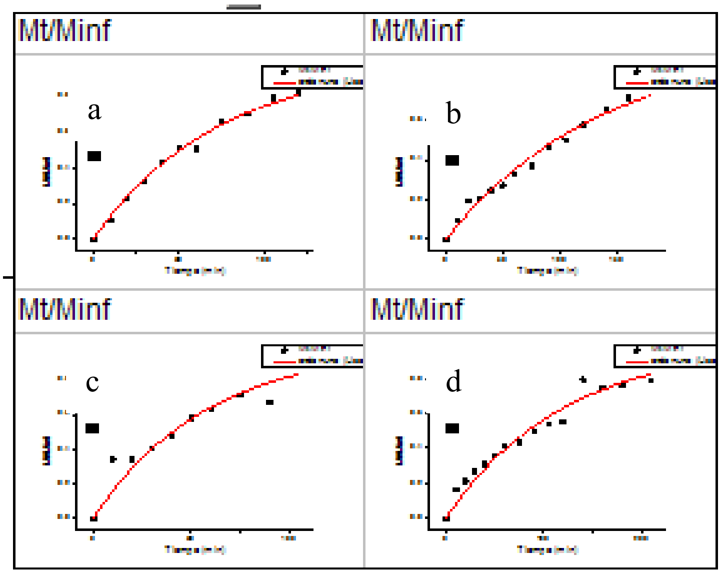

Figure S10. FO release profiles for tablets at pH 6: (a) T25a10 (4000-4500 ALU), (b) T15a10 (4000-4500 ALU), (c) T25a10 (500-900 ALU), (d) T15a10 (500-900 ALU)

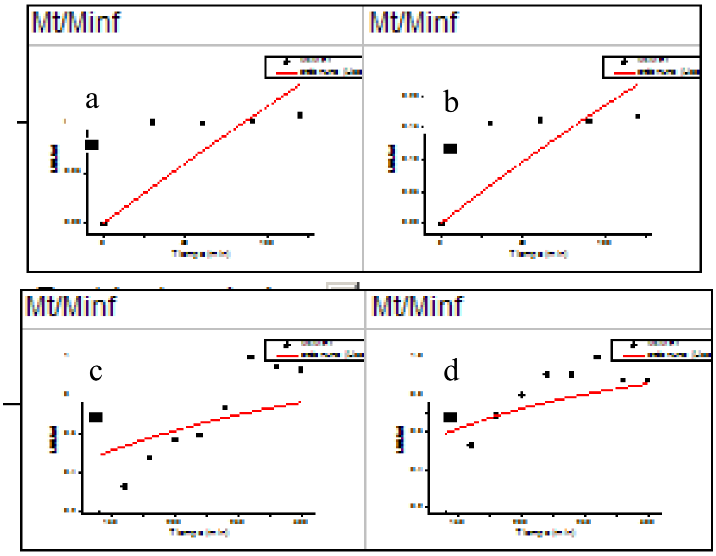

Figure S11. FO release profiles (4000-4500 ALU) from tablets with pH change: (a) T25a10 pH 1.2, (b) T15a10 pH 1.2, (c) T25a10 pH 6, (d) T15a10 pH 6

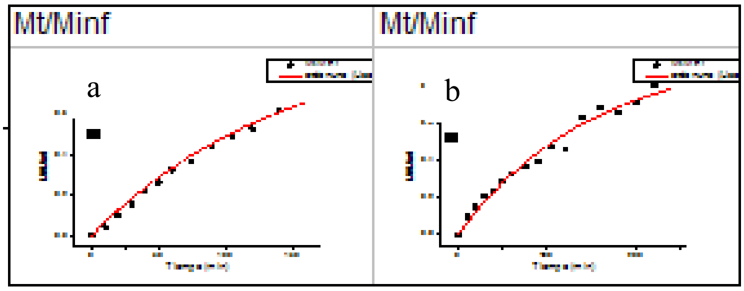

Figure S12. FO release profiles from gels at pH 6 (29450 ALU): (a) T25a10, (b) T15a10

Peppas-Sahlin (PS)

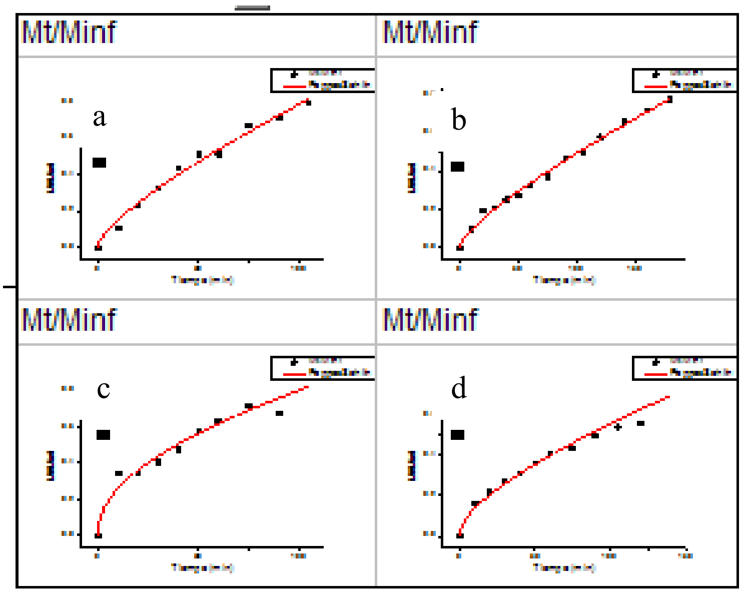

Figure S13. PS release profiles for tablets at pH 6: (a) T25a10 (4000-4500 ALU), (b) T15a10 (4000-4500 ALU), (c) T25a10 (500-900 ALU), (d) T15a10 (500-900 ALU)

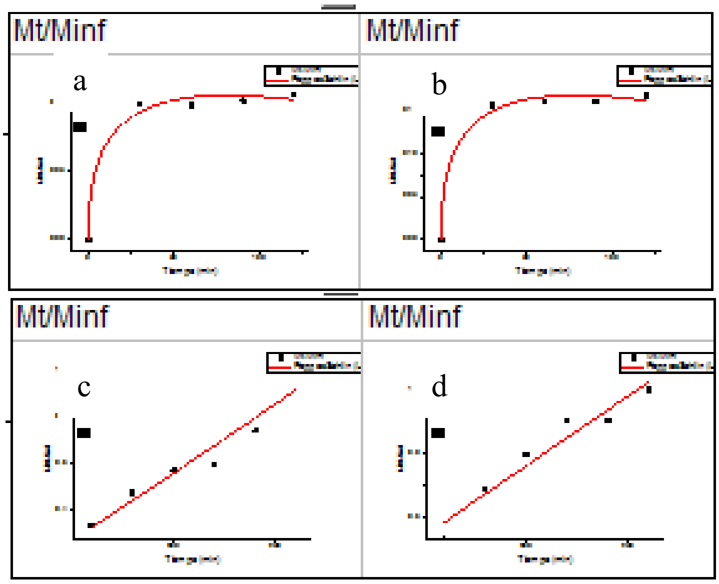

Figure S14. PS release profiles (4000-4500 ALU) from tablets with pH change: (a) T25a10 pH 1.2, (b) T15a10 pH 1.2, (c) T25a10 pH 6, (d) T15a10 pH 6

Hopfenberg (HF)

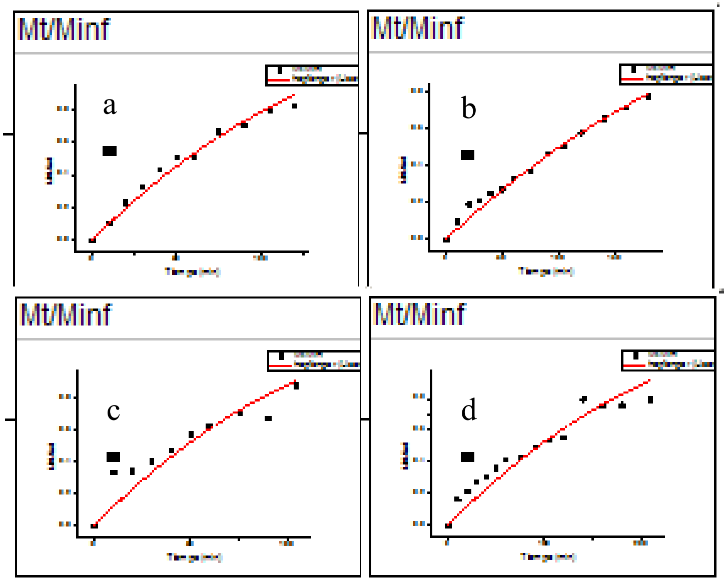

Figure S15. HF release profiles for tablets at pH 6: (a) T25a10 (4000-4500 ALU), (b) T15a10 (4000-4500 ALU), (c) T25a10 (500-900 ALU), (d) T15a10 (500-900 ALU)

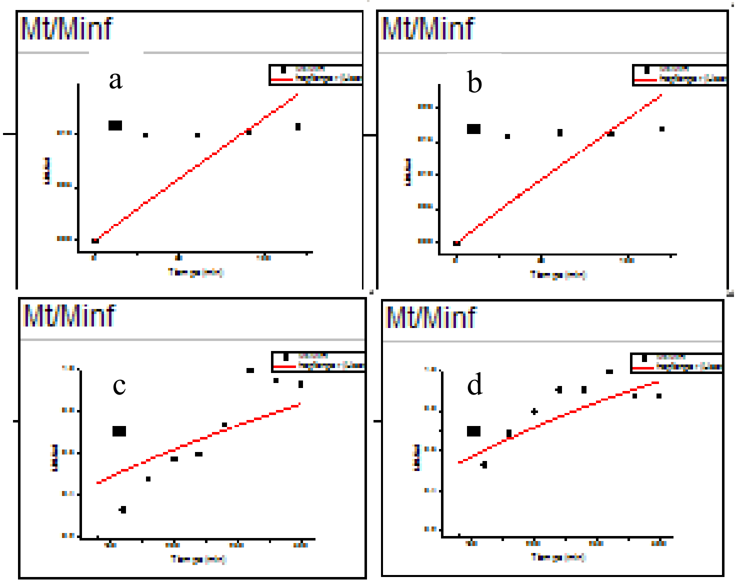

Figure S16. HF release profiles (4000-4500 ALU) from tablets with pH change: (a) T25a10 pH 1.2, (b) T15a10 pH 1.2, (c) T25a10 pH 6, (d) T15a10 pH 6
